# Herpes Simplex Virus Type 1 Infection Facilitates Invasion of *Staphylococcus aureus* into the Nasal Mucosa and Nasal Polyp Tissue

**DOI:** 10.1371/journal.pone.0039875

**Published:** 2012-06-29

**Authors:** XiangDong Wang, Nan Zhang, Sarah Glorieux, Gabriele Holtappels, Mario Vaneechoutte, Olga Krysko, Luo Zhang, Demin Han, Hans J. Nauwynck, Claus Bachert

**Affiliations:** 1 Upper Airways Research Laboratory, Department of Oto-Rhino-Laryngology, Ghent University Hospital, Ghent, Belgium; 2 Department of Otolaryngology Head and Neck Surgery, Beijing TongRen Hospital, Capital Medical University, Beijing, People’s Republic of China; 3 Laboratory of Virology, Faculty of Veterinary Medicine, Ghent University, Ghent, Belgium; 4 Laboratory of Bacteriology Research, Department of Clinical Chemistry, Microbiology and Immunology, Ghent University, Ghent, Belgium; 5 Key Laboratory of Otolaryngology Head and Neck Surgery (Capital Medical University), Ministry of Education, Beijing Institute of Otolaryngology, Beijing, People’s Republic of China; National Institutes of Health, United States of America

## Abstract

**Background:**

*Staphylococcus aureus* (*S. aureus*) plays an important role in the pathogenesis of severe chronic airway disease, such as nasal polyps. However the mechanisms underlying the initiation of damage and/or invasion of the nasal mucosa by *S. aureus* are not clearly understood. The aim of this study was to investigate the interaction between *S. aureus* and herpes simplex virus type 1 (HSV1) in the invasion of the nasal mucosa and nasal polyp tissue.

**Methodology/Principal Findings:**

Inferior turbinate and nasal polyp samples were cultured and infected with either HSV1 alone, *S. aureus* alone or a combination of both. Both in turbinate mucosa and nasal polyp tissue, HSV1, with or without *S. aureus* incubation, led to focal infection of outer epithelial cells within 48 h, and loss or damage of the epithelium and invasion of HSV1 into the lamina propria within 72 h. After pre-infection with HSV1 for 24 h or 48 h, *S. aureus* was able to pass the basement membrane and invade the mucosa. Epithelial damage scores were significantly higher for HSV1 and *S. aureus* co-infected explants compared with control explants or *S. aureus* only-infected explants, and significantly correlated with HSV1-invasion scores. The epithelial damage scores of nasal polyp tissues were significantly higher than those of inferior turbinate tissues upon HSV1 infection. Consequently, invasion scores of HSV1 of nasal polyp tissues were significantly higher than those of inferior turbinate mucosa in the HSV1 and co-infection groups, and invasion scores of *S. aureus* of nasal polyp tissues were significantly higher than those of inferior turbinate tissues in the co-infection group.

**Conclusions/Significance:**

HSV1 may lead to a significant damage of the nasal epithelium and consequently may facilitate invasion of *S. aureus* into the nasal mucosa. Nasal polyp tissue is more susceptible to the invasion of HSV1 and epithelial damage by HSV1 compared with inferior turbinate mucosa.

## Introduction

*Staphylococcus aureus*, a gram-positive bacterium commonly found as part of the normal microflora of the human skin, the upper respiratory tract (particularly the nares) and the intestinal tract, has been shown to cause a number of illnesses ranging from minor skin infections to life-threatening diseases such as pneumonia, meningitis, toxic shock, osteomyelitis, endocarditis, bacteremia, and autoimmune disorders.^1^ The mechanisms underlying *S. aureus* pathogenicity involve a variety of staphylococcal enterotoxins (SAEs) that act as superantigens [1], [2], capable of activating T cells and B cells. Several studies have indicated that SAEs play an important role in the pathogenesis of upper airways disease, particularly chronic rhinosinusitis and nasal polyposis [3]–[7], with more recent findings suggesting that SAEs may also be involved in the pathogenesis of asthma [8]–[10].

Despite evidence for the high colonisation rates of the airway mucosa by *S. aureus* [7],^7^ the mechanisms underlying the damage or invasion of the nasal mucosa by *S. aureus* are not clearly understood. A recent study by Corriveau and colleagues [11], employing the peptide nucleic acid-fluorescence *in situ* hybridization (PNA-FISH) technique, has demonstrated that *S. aureus* was present intramucosally and intracellularly in great quantities in the nasal mucosa of aspirin-sensitive patients with nasal polyps, but not in control subjects. Moreover, significant increases in Th2 markers (eosinophil cationic protein and total IgE) were correlated with the presence of specific IgE-antibodies against SAEs, suggesting that factors that modulate the release of SAEs are likely to play a role in intramucosal invasion by *S. aureus*.

As bacterial infections of the airways have often been shown to follow viral infections [12], we hypothesized that in patients with airway disease chronic invasion and colonization of the nasal mucosa by *S. aureus* may be a consequence of an interaction between specific viral and *S. aureus* components, which results in enhanced disruption of the nasal epithelium and basement membrane. To test this hypothesis, we have used an explant culture model of intact human turbinate tissue [13] and nasal polyp tissue to evaluate epithelial damage and intramucosal invasion by *S. aureus*, following infection with herpes simplex virus type 1 (HSV1). The choice of HSV1 as the infecting virus was based on its comparatively high global seroprevalence [14]–[17] and findings from recent studies, which have underlined the presence and possible role of HSV1 in upper airway disease: nearly 10% of human nasal polyps are infected with HSV1 [18], 2 out of 8 nasal mucosal samples taken at autopsy were positive for HSV1 [19], and HSV infection is a risk factor for nasal carriage of S. aureus in human immunodeficiency virus (HIV)-infected patients [20].

## Methods

### Nasal Turbinate Tissue

Inferior turbinate mucosal tissue was obtained from 10 patients scheduled for turbinate surgery due to septal deviations or turbinate hypertrophy and 7 nasal polyp tissue was obtained from endoscopic sinus surgeries at the department of Oto-Rhino-Laryngology, Ghent University Hospital. The patients were refrained from using oral or nasal corticosteroids for four weeks and antibiotics for two weeks before surgery. All patients provided written informed consent, and the ethics committees of the Ghent University Hospital approved the study.

### HSV1 Stocks

HSV1 (ATCC VR-733) was purchased from the American Type Culture Collection (ATCC; Rockville, MD, USA) and propagated to large quantities by infection of African green monkey kidney (Vero) cells (ATCC CCL-81; Rockville, MD, USA). The virus strains were passaged twice and diluted in serum-free medium to a final concentration of 10^7^ TCID_50_/ml. Equal amounts of RPMI medium 1640 (Invitrogen, Merelbeke, East Flanders, Belgium) and Dulbecco's Modified Eagle Medium (Invitrogen, Belgium) were used for all subsequent experiments involving infection of nasal turbinate tissue *ex-vivo.*

### *S. aureus* Stocks

*S. aureus* strain RN 6390 was chosen as the infecting strain in these studies because it has been well characterised; in particular it has shown to produce several important *S. aureus* exotoxins including hemolysin-A, -B, and -D and V8 protease, and is employed in several animal models of staphylococcal pathogenesis [21]–[23], and is labelled with the gene for green fluorescence protein (GFP) [22]. A stock of *S. aureus* strain RN 6390 (Provided by Prof. C. von Eiff, Munster, Germany) was grown in Trypticase Soy-Yeast Extract Broth (TSB; BD Biosciences, Erembodegem, East Flanders, Belgium) for 24 h at 37°C, on the day prior to use as an infecting agent. At the end of the incubation, the concentration of the *S. aureus* in the broth was assessed according to the optical density (OD) of the suspension, measured using a Beckman DU640B spectrophotometer (Beckman Instruments, Fullerton, CA, USA), and the suspension was centrifuged at 2500x g for 5 min. The medium was decanted and the *S. aureus* pellet was washed three times by resuspension and centrifugation in equal volume of phosphate buffered saline (PBS). The washed *S. aureus* pellet was resuspended in serum-free medium to a concentration of 20×10^6^ CFU/ml for use in subsequent experiments involving infection of nasal turbinate tissue *ex-vivo*. Based on former ex vivo experiments in cultured epithelial cells described in the literature [24], [25], we used bacterial concentrations of 10^7^–10^8^ CFU/ml and prepared pilot experiments to finally choose a concentration of 20×10^6^ (2x10^7^) CFU/ml as sub-optimal effective dose, which did not overgrow the mucosa.

### Culture and Infection of Nasal Turbinate and Nasal Polyp Explants with HSV1± *S. aureus*

Nasal turbinate and nasal polyp tissue obtained from each patient following surgery was immediately washed three times with serum-free medium supplemented with antibiotics (50 IU/mL penicillin (Invitrogen, Belgium) and 50 µg/mL streptomycin (Invitrogen, Belgium) and cultivated as described previously [13]. The washed tissue explant was cut into smaller cubes approximately 25 mm^2^ in size. Eight nasal tissue cubes of each turbinate or nasal polyp explants were used for further investigation which were divided into four equal groups of two cubes each (Group 1 =  HSV1 infection group; Group 2 =  *S. aureus* infection group; Group 3 =  HSV1+ *S. aureus* infection group; and Group 4 =  control, non-infection group) (Table S1, online data supplement of tissue culture protocol). Each cube was placed with the epithelial surface upwards on a sterile fine-meshed gauze in a 6-well tissue-culture plate (Falcon, BD Biosciences, Erembodegem, East Flanders, Belgium) and 3 ml serum-free medium supplemented with antibiotics was added to each well to create an air-liquid interface. All tissue cubes were conditioned as explant cultures by incubation for 24 h at 37°C in 5% CO_2_ in air atmosphere (culture stage 1), and then transferred to a 24-well tissue-culture plate (Falcon, BD Biosciences, Belgium). Groups 1 and 3 tissue cubes were inoculated with 1.0 ml inoculum containing 10^7^ TCID_50_ of HSV1, and 1.0 ml of serum-free medium was added to the tissue cubes in Groups 2 and 4 as mock-condition, all tissue cubes were incubated (culture stage 2) for 1 h at 37°C in 5% CO_2_ in air atmosphere. All tissue cubes were washed three times, transferred onto a sterile fine-meshed gauze and incubated in a 6-well tissue-culture plate (culture stage 3) for either 24 h or 48 h under air-liquid interface culture conditions as before. At the end of each incubation period, the tissue cubes were transferred to a 24-well tissue-culture plate. 1.0 ml of *S. aureus* infection medium containing 20×10^6^ CFU was added to the cubes in Groups 2 and 3 and 1.0 ml of serum-free medium to the tissue cubes in Groups 1 and 4, and all tissue cubes were incubated for 2 h at 37°C in 5% CO_2_ in air atmosphere (culture stage 4). Following incubation, all tissue cubes were transferred to a 6-well tissue-culture plate for a final 24 h incubation period (culture stage 5) on fine-meshed gauze at an air-liquid interface. At the end of the culture, the tissue cubes were weighed and snap-frozen in liquid nitrogen and stored at −80°C, until further assessment by immunohistochemistry.

### Immunofluorescence and Hematoxylin Staining

Frozen tissue cubes were assessed for nasal epithelial and basement membrane damage and viral and bacterial invasion by hematoxylin- and immunofluorescence-staining, respectively. Cryosections (5 µm thickness) were prepared using a Shandon Cryotome® (Thermo, Runcorn, Cheshire, UK). The first and last 1 mm of the tissue cubes were discarded. Every 300 µm, two sections were prepared to evaluate HSV1 and SA invasions with immunofluorescence staining, and nasal epithelial damage with hematoxylin staining. 10 sections of each tissue cube were processed for staining.

### Immunofluorescence Staining for Evaluation of HSV1/*S. aureus* Invasion

Briefly, the tissue sections were fixed in 3% paraformaldehyde (Sigma-Aldrich, Bornem, Antwerp, Belgium) for 25 min at room temperature and then washed twice with PBS. The sections were treated with 0.1% triton-X 100 (Sigma-Aldrich, Belgium) for 2 min and washed twice with PBS, incubated for 10 min at room temperature in the presence of 10% Normal Goat Serum (NGS) (Invitrogen, Belgium), to block nonspecific binding sites. At the end of this incubation, the NGS was decanted and the sections were incubated for 1 h at 37°C in the presence of mouse anti-HSV1-gD antibodies (Santa Cruz, Heidelberg, Baden-Württemberg, Germany) (100 µg/ml, 1∶100 in 10% NGS) or mouse IgG2 as an isotype specific negative control antibody (Dako, Glostrup, Region Hovedstaden, Denmark). Following three washings with PBS, the sections were incubated for a further h at 37°C in the presence of goat anti-mouse-Texas Red antibodies (Molecular Probes, Invitrogen, Belgium) (2 mg/ml, 1∶50 in 10% NGS). At the end of this incubation, the sections were washed three times with PBS and once with ultrapure water and dried at room temperature, prior to being mounted with glycerin-DABCO (Janssen Chemica, Beerse, Antwerp, Belgium).

### Hematoxylin Staining for Evaluation of Epithelial Damage

Slides were stained with hematoxylin (Sigma-Aldrich, Belgium) for 1 min and washed with tap water for 5 min. The slides were mounted with Aquatex (Merck KGaA, Darmstadt, Hessen, Germany).

### Evaluation of HSV1/*S. aureus* Invasion and Epithelial Damage

Immunofluorescence-stained slides were evaluated for viral and bacterial invasion by viewing at ×630 magnification using a fluorescence microscope (Axioplan 2, Carl Zeiss, Gottingen, Lower Saxony, Germany), and hematoxylin-stained slides were evaluated for epithelial damage by viewing at ×400 magnification using a light microscope (CX40RF200, Olympus, Tokyo, Japan). All stained slides were evaluated by two independent observers (LF and MJ), who were blinded to the tissue-treatment protocol and assessed the entire epithelium in each section by viewing up to 8–10 adjacent fields. To exclude penetration from the non-epithelial sides, we omitted the outer edges of the tissue cubes and only took specific invasion patterns from the epithelium to the lamina propria into account. HSV1 and *S. aureus* invasion in each field was graded on a 5-point scale (0 = epithelium not infected, 1 = epithelium superficially infected, 2 = basal cells infected, 3 = basement membrane and HSV1 and/or *S. aureus* colocalisation, HSV1 or *S. aureus* do not penetrate the basement membrane, 4 = HSV1 or *S. aureus* penetrated the basement membrane into the lamina propria); whereas epithelial damage was graded on a 4-point scale (0 = no damage, epithelium totally preserved; 1 = focal and superficial damage, superficial cells desquamate; 2 = epithelial damage involving basal cells, basal epithelial cells partly detached; 3 = epithelium severely damaged, loose). The mean of total scores in the ten sections on each slide was used as the final epithelial damage or invasion score for each explant.

### Statistical Analysis

All data are expressed as mean ± SEM for each treatment group and differences between the groups were compared by the analysis of variance (ANOVA) assessed using the SPSS version 11.5 software (SPSS Inc, Chicago, USA). Correlations between HSV1 and/or *S. aureus* invasion scores and epithelial damage scores were assessed using MEDCALC version 11.3.3 software (F.Schoonjans, Mariakerke, Belgium). P-values <0.05 were considered to be significant.

## Results

### Patient Characteristics

Clinical characteristics, skin prick test and IgE data of all patients are summarized in Table 1. The two groups were comparable in terms of age, female/male ratio, asthma, aspirin intolerance, chronic obstructive pulmonary diseases (COPD) and atopy (positive skin prick tests or Phadiotop result). Patients with Churg-Strauss Syndrome or other immune disorders were excluded from this study.

**Table 1:** Patient characteristics.

	Inferior turbinategroup	Nasal polypsgroup
No.	10	7
Age(y), median	30.4(17.4–44.8)	48.5(17.4–64.8)
Female/male, sex	1/9	0/7
Asthma	1/10	2/7
Aspirin intolerance	0/10	1/7
COPD	0/10	1/7
Previous surgery	0/10	1/7
Skin pricktests/specific IgE	1/10	1/7

**Figure 1:**
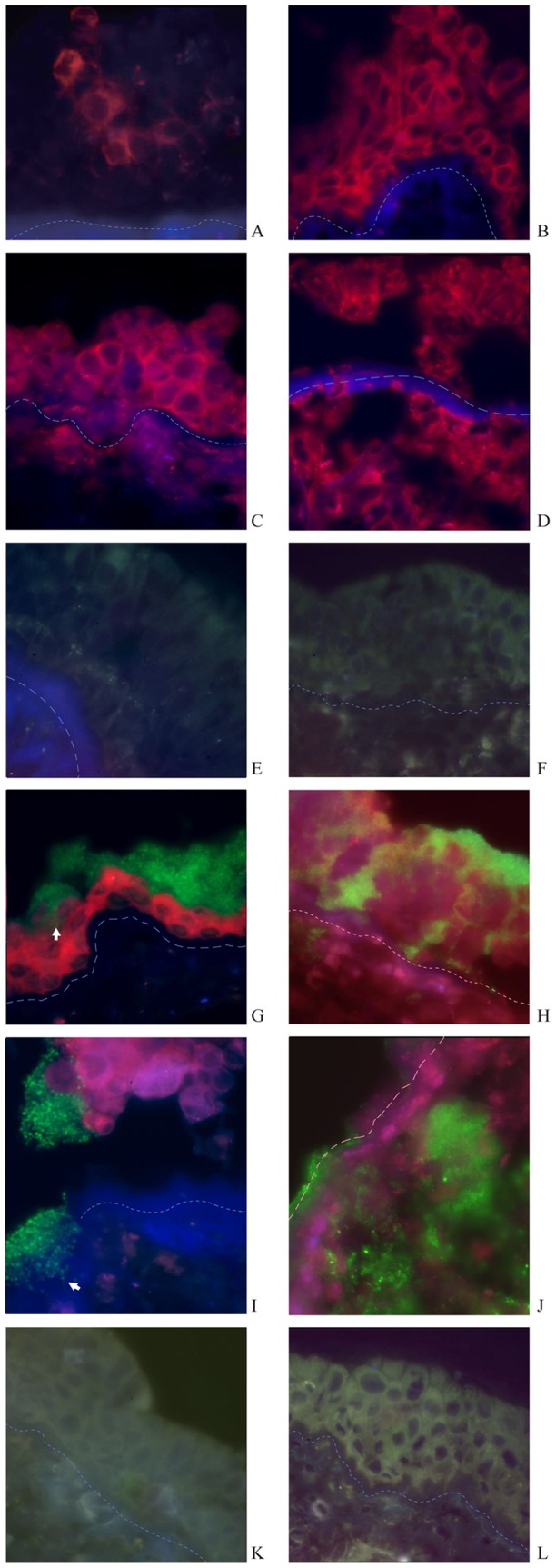
Immunofluorescence-stained sections of nasal mucosa of inferior turbinate (IT) and nasal polyp tissue samples (NP). HSV1 infection: After 24 h HSV1+24 h medium incubation, HSV1 infects outer epithelial cells of IT focally, without reaching the basement membrane and results in loss of epithelial integrity (A). In contrast, HSV1infected the whole epithelium of NP after 24 h of HSV1 incubation (B). After 48 h HSV1+24 h medium incubation, HSV1 infects basal epithelial cells, and leads to loss of epithelial structure partly, penetration of the basement membrane and invasion of the virus into the lamina propria of IT (C). HSV1 infection leads to the loss of epithelial structures and invasion of the virus into the lamina propria of NP diffusively (D). *S. aureus* infection: After 24 h medium+24 h *S. aureus* incubation, *S. aureus* does not infect or invade the epithelium of IT and NP (E,F). HSV1+*S. aureus* infection: After 24 h HSV1+24 h *S. aureus* incubation, *S. aureus* forms clusters and attaches to the residual epithelial cells infected by HSV1, with *S. aureus* penetration of epithelium through inter-epithelial cell spaces (white arrow) (G). In contrast, cluster-forming *S. aureus* attached to epithelial cells of NP when infected by HSV1and penetrated the epithelium down to basal cells widely; planctonic bacteria invaded into the lamina propria after 24 h combined HSV1and *S. aureus* incubation (H). After 48 h HSV1+24h *S. aureus* incubation, clusters of *S. aureus* adhere to HSV1-infected epithelial cells and basement membrane of IT, with subsequent shedding of epithelial cells and penetration of the basement membrane (white arrow) (I). Whereas both HSV1 and *S. aureus* invade into the lamina propria of NP diffusively, with loss of epithelium(J). Control: The epithelium is intact and undamaged after 96 h totally in non-infected control tissues explants of IT and NP (K,L). (All slides viewed at ×630 magnification; red fluorescence demonstrates the presence of HSV1 and green fluorescence the presence of *S. aureus* in the nasal mucosa; dotted line indicates the basement membrane. The isotype control staining for HSV1 was completely negative and is not presented here.).

### HSV1 and *S. aureus* Invasion of Nasal Mucosa

Figure 1 shows invasion of the nasal mucosa by HSV1 and *S. aureus*. Infection of the nasal turbinate mucosal tissue with HSV1 alone led to focal infection of outer epithelial cells (red fluorescence) with distribution up to the basement membrane and damage of epithelial structural integrity after 48 h (24 h pre-incubation with HSV1, followed by 24 h mock-treatment) in several cases (Figure 1A). Incubation of the tissue for 72 h following inoculation with HSV1 (48 h pre-incubation with HSV1, followed by 24 h mock-treatment) led to infection of basal epithelial cells, followed by loss of the epithelium and subsequent invasion of HSV1 into the lamina propria (Figure 1C). In contrast, HSV1 invaded into the whole epithelium of nasal polyp tissue causing partial damage (Figure 1B) after 48 h and into the lamina propria through the basement membrane with significant damage of the epithelium after 72 h (Figure 1D).

Incubation of the nasal mucosa in the presence of *S. aureus* alone did not affect the epithelium and there was little or no attachment of the bacterium to the epithelium of nasal turbinate mucosa and nasal polyp tissue (Figure 1E and 1F). However, incubation of the nasal mucosa with *S. aureus* following incubation with HSV1 led to formation and attachment of *S. aureus* clusters (green fluorescence) to the HSV1-infected residual epithelial cells following 24 h of HSV1 infection (Figure 1G). After 48 h of HSV1 incubation, followed by 24 h incubation with *S. aureus*, clusters of *S. aureus* adhered to HSV1-infected epithelial cells and to the basement membrane with invasion into the basement membrane (Figure I). In a co-infection model of nasal polyp tissue with HSV-1, *S. aureus* invaded into the epithelium through the intercellular spaces and occasionally reached the basement membrane after 24 h of incubation (Figure 1H); this co-infection resulted in marked invasion of the lamina propria by both HSV1 and *S. aureus* after 72 hours accompanied by a significant loss of epithelium (Figure 1J).

In contrast, the epithelium in non-infected (control) tissue of nasal turbinate mucosa and nasal polyp was intact and undamaged after 96 h of incubation under similar culture conditions (Figure 1K and 1L).

**Figure 2:**
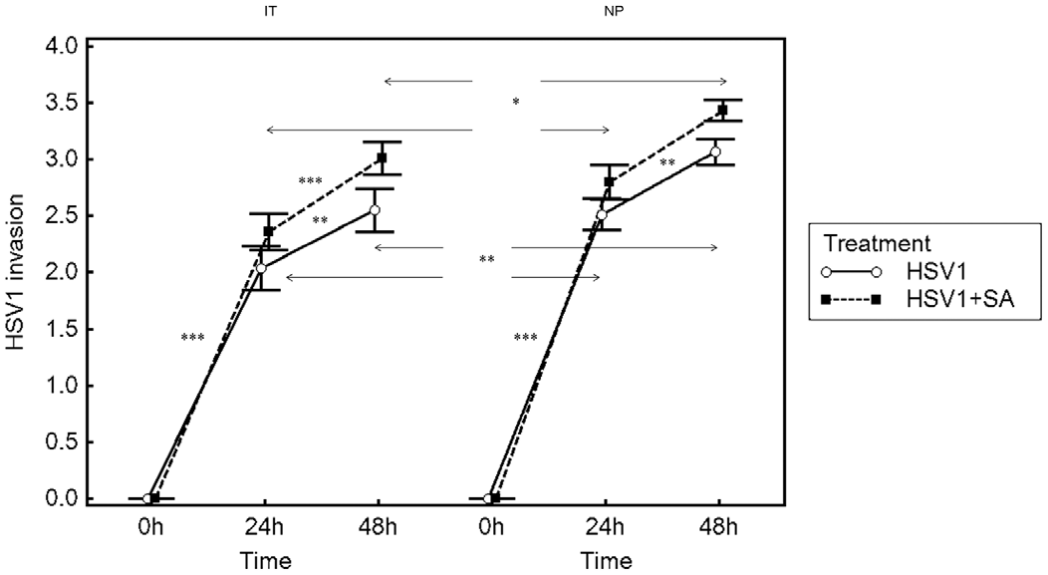
Invasion of nasal mucosa of inferior turbinate (IT) and nasal polyp tissue samples (NP) by HSV1. Results are presented as mean scores ± SEM for IT from 10 patients and NP from 7 patients. Arrows represent differences between HSV1-infection and HSV1+*S. aureus*-infection groups or differences between IT group and NP groups. *P<0.05, **P<0.01 and ***P<0.001.

Evaluation of nasal turbinate mucosal invasion scores for HSV1 demonstrated that the invasion of HSV1 was significantly increased from baseline at 48 and 72 h post inoculation (pi) (after 24 h mock-treatment following 24 and 48 hpi) (p<0.001 for both time points), and that invasion was significantly greater after 48 hpi compared to invasion after 24 hpi in HSV1 infection group (p<0.01) and in HSV1+SA infection group (p<0.001). Furthermore, the invasion of HSV1 was not significantly altered by subsequent infection of the nasal mucosa with *S. aureus* (Figure 2). The invasion scores of HSV1 in nasal polyp tissue were similar to turbinate mucosa, but the scores at 48 and 72 h were significantly higher than those of turbinate mucosa (p<0.05 and p<0.01 respectively) (Figure 2). We checked interrater reliability using the Fleiss k coefficient, which was 0.72, showing a good agreement between the two observers.

**Figure 3:**
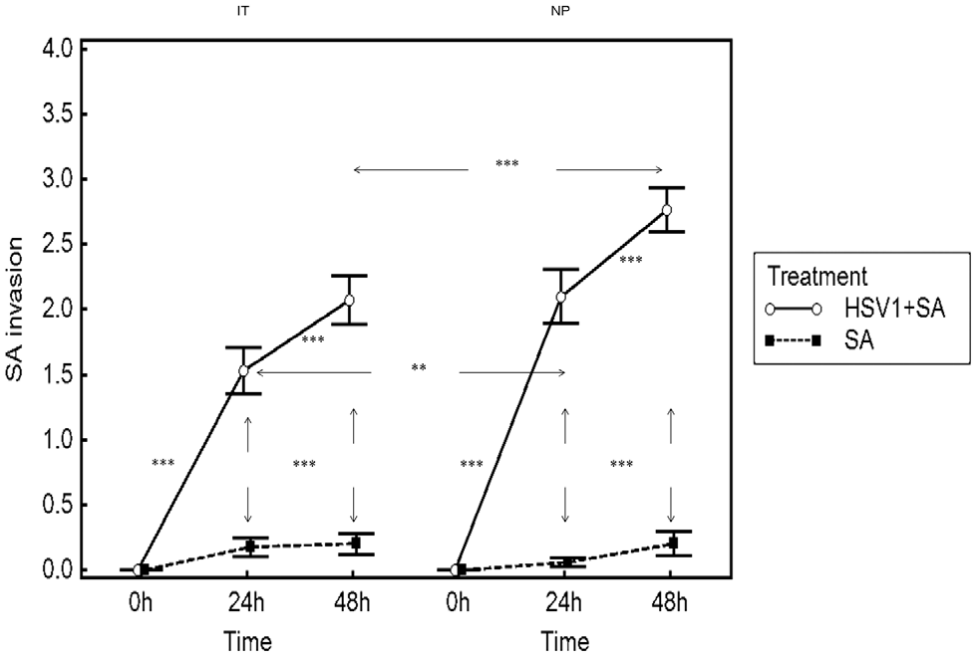
Invasion of nasal mucosa of inferior turbinate (IT) and nasal polyp tissue samples (NP) by *S. aureus*. Results are presented as mean scores ±SEM for nasal turbinate samples from 10 patients and NP from 7 patients. Arrows represent differences between *S. aureus*-infection and HSV1+*S. aureus*-infection groups or differences between IT group and NP groups. ** *P*<0.01,*** *P*<0.001.

Similarly, evaluation of the nasal turbinate mucosal invasion score for *S. aureus* demonstrated that in the absence of prior infection with HSV1, the invasion of the nasal mucosa was not significantly altered from baseline following either 24 or 48 h post mock-treatment (Figure 3). Infection of the nasal mucosa with HSV1 prior to infection with *S. aureus*, however, significantly increased the *S. aureus* invasion scores from baseline following 24 and 48 h post HSV1-infection (p<0.001 for both time points). The invasion of *S. aureus* was also significantly greater following 48 h post HSV1-infection compared to 24 h post HSV1-infection (p<0.05) (Figure 3). The invasion scores of *S. aureus* in nasal polyp tissue were also similar to those of nasal turbinate musosa, but the scores of 48 and 72 h after co-infection were significantly higher than those of turbinate mucosa (p<0.01 or p<0.001, respectively) (Figure 3).

### Epithelial Damage and Correlation with Invasion by HSV1 and *S. aureus*

**Figure 4:**
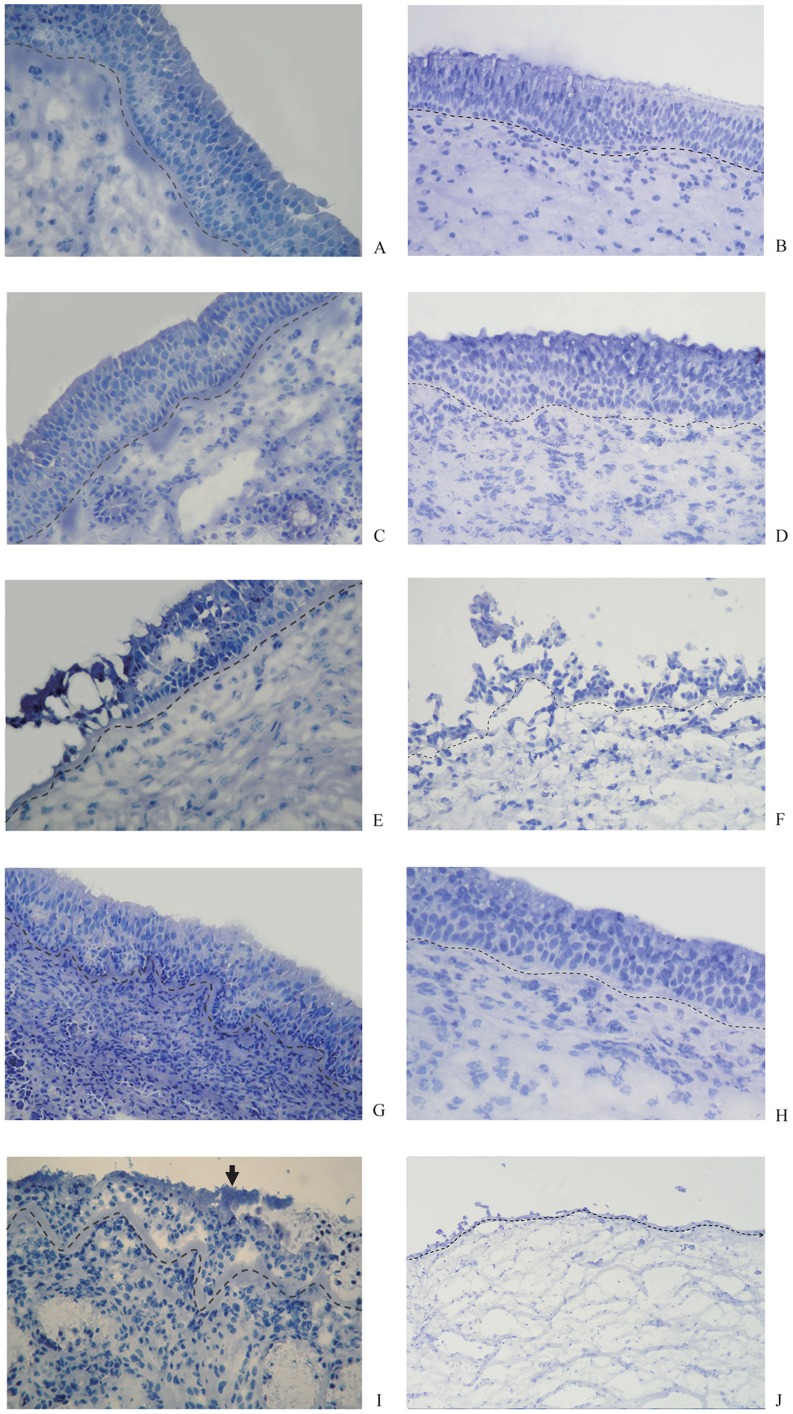
Hematoxylin-stained sections of inferior turbinate (IT) and nasal polyp tissue samples (NP). The epithelium and basement membrane (dotted line) are intact and undamaged at baseline of IT (A) and NP (B) respectively and after 96 h totally incubation in uninfected control explants of IT (C) and NP (D), respectively. Extensive infection and damage to the epithelium is seen in the explants after 48 h HSV1+24h medium of IT (E) and NP (F) respectively, but not in the explants of IT (G) and NP (H) respectively after 48h medium+24 h *S. aureus* incubation. Combined HSV1 and *S. aureus* infection leads to adherence of *S. aureus* clusters to the epithelium (arrow) and partial damage of the epithelium of IT after 24 h HSV1+24 h *S. aureus* incubation (I), and complete loss of the epithelium of NP after 24 h HSV1+24 h *S. aureus* incubation (J). (All slides viewed at ×400 magnification.)

**Figure 5:**
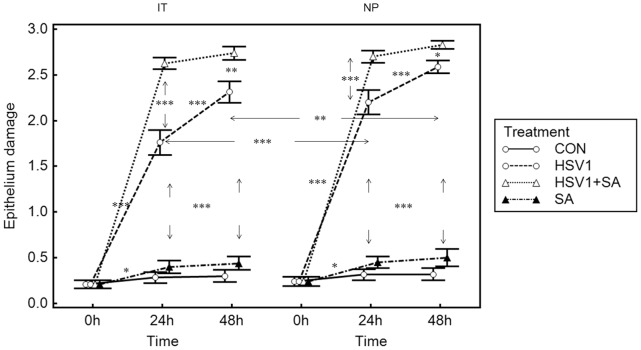
Epithelium damage in nasal mucosa. Results are presented as mean±SEM scores for IT from 10 patients and NP from 7 patients; *P<0.05, **P<0.01, ***P<0.001 between time points. Control means no infection. Arrows indicate intergroup significance.

Hematoxylin-staining of nasal turbinate mucosa and nasal polyp tissue explants indicated that the tissue-culture conditions employed in the present study were suitable for the preservation of epithelial integrity over a period of 96 h (Figure 4B), as demonstrated by the presence of an intact and normal epithelium in non-infected control cultures, that was comparable with baseline (Figure 4A). Indeed, assessment of epithelial damage scores demonstrated that the scores were not significantly different in control culture conditions, where explants were first mock-treated with medium for 24 or 48 h and subsequently incubated with medium (mock) for another 24 h until sampling, compared to baseline scores (Fig 5).

Although infection of the nasal mucosal explants with HSV1 (Figure 4C), but not *S. aureus* (Figure 4D), led to extensive epithelial damage after respectively 24 h of mock- and *S. aureus*-treatment following 48 h of respectively HSV1- and mock-treatment, the damage to the epithelium was compounded (Figure 4E) and complete (Figure 4F) when the nasal mucosa was infected with HSV1 during 24 and 48 h respectively followed by 24 h of infection with *S. aureus*. Assessment of epithelial damage scores further confirmed these histological findings and demonstrated that the scores were significantly higher for HSV1 with and without *S. aureus*-infected explants, compared to non-infected control explants and *S. aureus* only-infected explants (Fig 5). Moreover, the damage was significantly greater at 72 hpi (48 h of HSV1+24 h of mock treatment) compared to 48 hpi (24 h of HSV1+24 h of mock treatment) in HSV1 only-infected explants, but not in co-infected explants where 24h infection with *S. aureus* followed 48 h pre-infection with HSV1 compared to *S. aureus*-infection following 24 h of pre-infection with HSV1. In co-infected explants, high damage scores were already reached for *S. aureus*-infection following 24 h of HSV1-infection.

The epithelial damage patterns of nasal polyp tissue after infection with HSV1, *S. aureus*, or both resemble those of nasal turbinate mucosa; however, the damage scores after infection for 24 and 48 h were significantly higher than those in turbinate mucosa (Fig 5).

Evaluation of correlations between epithelial damage scores and HSV1 invasion scores demonstrated significant correlations in both the HSV1-infected explants of turbinate mucosa (*R* = 0.74, *P*<0.001) and nasal polyp tissue (*R* = 0.706, *P*<0.001) and the HSV1+*S. aureus*-infected explants of turbinate (*R* = 0.63, *P*<0.001) and nasal polyp tissues (*R* = 0.641, *P*<0.001).

## Discussion

*S. aureus* is both a human commensal and a frequent pathogen of clinically important infections; with about one-third of the population being a permanent carrier of *S. aureus* [1]. Although *S. aureus* has been detected in nasal polyp tissue [11], the mechanisms underlying *S. aureus* invasion into the nasal mucosa are not clearly understood to date. As bacterial infection has been demonstrated often to be secondary to viral infection [8], it is tempting to speculate that an interaction between viral and *S. aureus* infections is likely, at least in part, to be responsible for the invasion of *S. aureus* into the nasal mucosa of these patients.

We used an *ex-vivo* explant tissue culture model [13] to investigate the effect of prior infection with HSV1, a prevalent virus [14]–[18], on subsequent damage and invasion by *S. aureus* in human nasal mucosal tissue. Our study demonstrated that prior infection of human nasal explants with HSV1 in the absence or presence of subsequent *S. aureus-*infection led to focal infection of epithelial cells after mock/*S.aureus*-treatment following 24 h of pre-infection with HSV1 and loss or damage of the epithelium accompanied by invasion of HSV1 into the lamina propria following 48 h of pre-infection with HSV1. In contrast, invasion by *S. aureus* into the nasal mucosa was noted in explants subjected to a prior 48 h of HSV1-infection followed by a subsequent *S. aureus-*infection, but not in explants subjected to only *S. aureus*-infection or in non-infected control explants. Evaluation of graded mean scores for the invasion of HSV1 and *S. aureus* into the nasal mucosa confirmed the immunohistological observations and demonstrated that HSV1 invasion was significantly increased from baseline in HSV1-infected explants independent of *S. aureus*, whereas invasion by *S. aureus* was significantly increased from baseline only after HSV1 pre-incubation. Epithelial damage and invasion of the mucosa by HSV1 were significantly more extensive in nasal polyp compared to inferior turbinate tissues, and consequently, mucosal invasion by *S. aureus* of nasal polyp tissues was increased vs. inferior turbinate samples.

Our findings of HSV1 invasion into the lamina propria are in line with Glorieux et al [13]. However, important to remark is that the authors observed HSV1 penetration through the basement membrane from 16 hpi onwards, with 100% of the plaques penetrating the basement membrane from 24 hpi. This could be explained by the fact that Glorieux et al [13]. evaluated serial sections covering the whole plaque of 10 different plaques for 3 different persons, whereas in the present study, 10 sections were evaluated with 2 sections processed every 300 µm. Herpesvirus invasion into the lamina propria was shown previously for animal viruses, such as pseudorabies virus (PRV) [26] and bovine herpesvirus 1 (BHV1) [27], which show plaquewise spread through the basement membrane. Equine herpesvirus 1 (EHV1) plaques remain confined to the epithelium and EHV1 invades below the basement membrane via EHV1-infected single mononuclear cells [28].

Similarly, graded mean scores for epithelial damage scores were significantly higher for HSV1 infected explants independent of *S. aureus*, compared with control explants or *S. aureus* infected explants; epithelial damage scores significantly correlated with HSV1-invasion scores. Collectively these findings suggest that HSV1-infection may facilitate the subsequent invasion and colonisation of the nasal mucosa by *S. aureus*; possibly by breaking down the intact epithelium and basement membrane that normally acts as a barrier to *S. aureus.* Similar to rhinovirus [29], [30] and adenovirus [31], HSV1 could disrupt cell junctions as late HSV replication is associated with disruption of cell junctions. However, the viral factors contributing to this disruption are unknown so far [32].

To our knowledge this is the first study to investigate the impact of HSV1 infection on the invasion of *S. aureus* into human airway mucosa using explant cultures. A small number of other studies have reported different aspects of viral infection in human nasal tissue cultures [33]–[38]; however, studies describing possible interactions between viral and bacterial infections/invasion are limited. Read and colleagues [33] investigated the effect of prior influenza B virus-infection on subsequent attachment and penetration of serogroup B *Neisseria meningitides* in nasopharyngeal explants from 19 patients. The authors demonstrated that bacterial association with the surface of explants was time dependent over 24 h of infection, however, the virus did not positively or negatively influence bacterial attachment to or penetration of explant mucosa compared to uninfected controls. Moreover, the proteins involved in the attachment of *N. meningitides* to the epithelial surface were lost over a period of 72 h in the presence of influenza B virus. A recent study by Wang and colleagues [39] demonstrated that prior rhinovirus serotype 16 (RV-16) infection significantly increased gene and protein expression for specific bacterial adhesion proteins in primary human nasal epithelial cell cultures. However, there was a differential attachment of *S. aureus*, *Streptococcus pneumoniae*, and *Hemophilus influenzae* to the epithelial cells, suggesting species-differences in the mechanisms underlying bacterial adhesion and/or epithelial invasion. In a separate study, these authors further demonstrated that although RV-induced expression of bacterial adhesion molecules was not augmented by incubation with staphylococcal enterotoxins A and B, these molecules lead to a dose-dependent replication of RV in epithelial cell cultures [40], suggesting that there may be interdependency between viruses and bacteria with respect to growth, invasion, or colonisation of nasal mucosa.

In the present study, we found that *S. aureus* invaded the deeper epithelial cells and lamina propria only after the mucosal explants were infected with HSV1, leading to epithelial damage, but not in the absence of prior HSV1-infection; in which case there was only occasional attachment of *S. aureus* to the surface of epithelium. Furthermore, in the case of HSV1 pre-incubation, *S. aureus* attached to the surface of the denuded basement membrane and formed a thick layer. These observations are in accordance with others showing that *S. aureus* attached to damaged or remodelled, but not to intact airway epithelium [41], [42]. Following damage to the epithelium, it is possible that *S. aureus* could readily adhere to basement membrane matrix proteins (collagen types I, IV and VI, fibronectin and laminin) [41], [43]–[46] before penetrating the basement membrane and colonising the lamina propria. Interestingly, the epithelial damage scores of the HSV1+*S. aureus* infected explants were significantly higher than those of the HSV1 infected explants. Recent findings have shown that staphylokinases of *S. aureus* may activate matrix metalloproteases (MMPs) that may degrade collagen [47], and MMP9 can cleave airway epithelial cell-cell junctions and trigger cell death [48], which indicate that products of *S. aureus* attaching to epithelial cells and basement membrane may exacerbate the damage of epithelium and basement membrane after HSV1 had induced the attachment of *S. aureus*.

In the present study, we show that HSV1 has an increased invasive ability into nasal polyp tissue accompanied with more serious damage effect of epithelium compared with nasal turbinate mucosa. It was demonstrated before that nasal polyp tissue is susceptible to influenza virus infection, associated with an increased expression of α2,3- and α2,6-linked sialic acid receptor [49]. Wark et al and Contoli et al demonstrated that epithelium of asthma subjects was defective in the production of interferon-β (IFN-β) and IFN-λ in response to rhinovirus [50], [51]; we may assume that the same mechanisms are valid in nasal polyps, characterized by a Th2 inflammation similar as asthma.

In summary, our study has indicated that the human nasal explant culture model is a suitable and reliable means to investigate the role of HSV1 and possibly other respiratory viruses in modulating the invasion by *S. aureus* of the airway mucosa. This ex-vivo human mucosal model allows comparing healthy with diseased mucosa, elucidating the underlying mechanisms of mucosal invasion, as the normal cell-cell contacts, the crucial cell-extracellular matrix contacts and consequently, the three-dimensional structure of the tissue are preserved. Using this model, we have demonstrated that HSV1 infection significantly damages the nasal epithelium and induces the attachment of *S. aureus*, thus facilitating the invasion of *S. aureus* into nasal mucosa. Nasal polyp tissue is more susceptible to the invasion of HSV1 and epithelial damage by HSV1 compared with inferior turbinate mucosa. We here studied the facilitating role of HSV1 for the acute invasion of *S. aureus* into the mucosa and used GFP-labeled *S. aureus*. This allows differentiating between preexisting colonization, either acute or chronic, and experimental exposure to *S. aureus*. Future studies using this model are planned to address the detailed mechanisms involved in HSV1 and *S. aureus* invasion of nasal mucosa and the roles of HSV 1 and *S. aureus* in the pathogenesis of nasal polyps.

## Supporting Information

Table S1 **Study protocol for tissue culture and HSV1±** ***S. aureus*** **(SA) infection stages.**
(DOCX)
