# Supplementary material for: Herpes Simplex Virus Type 1 Infection Facilitates Invasion of Staphylococcus aureus into the Nasal Mucosa and Nasal Polyp Tissue
Source: PLoS One. 2012 Jun 29;7(6):e39875. doi: 10.1371/journal.pone.0039875 (PMC3387208; doi:10.1371/journal.pone.0039875)
Supplement: Table S1 — Study protocol for tissue culture and HSV1± S. aureus (SA) infection stages. (DOCX) [file pone.0039875.s001.docx]

**Table S1.** Study protocol for tissue culture and HSV1 ± *S. aureus* (SA) infection stages

| **Nasal tissue-culture stage** | **Group 1**  **(HSV1 infected)** | **Group 2**  **(SA infected)** | **Group 3**  **(HSV1+SA infected)** | **Group 4**  **(Control)** |
| --- | --- | --- | --- | --- |
| ***1***  ***2***  ***3***  ***4***  ***5*** | Incubation overnight  HSV1 infection for 1h  Incubation for **24/48h**  In medium for 2h  Incubation for 24h | Incubation overnight  In medium for 1h  Incubation for **24/48h**  SA infection for 2h  Incubation for 24h | Incubation overnight  HSV1 infection for 1h  Incubation for **24/48h**  SA infection for 2h  Incubation for 24h | Incubation overnight  In medium for 1h  Incubation for **24/48h**  In medium for 2h  Incubation for 24h |
